# Effects of Soil Aggregate Stability on Soil Organic Carbon and Nitrogen under Land Use Change in an Erodible Region in Southwest China

**DOI:** 10.3390/ijerph16203809

**Published:** 2019-10-10

**Authors:** Man Liu, Guilin Han, Qian Zhang

**Affiliations:** 1 Institute of Earth Sciences, China University of Geosciences (Beijing), Beijing 100083, China; lman@cugb.edu.cn; 2 School of Water Resources and Environment, China University of Geosciences (Beijing), Beijing 100083, China; zhangqian9@cugb.edu.cn

**Keywords:** soil nutrients, agricultural abandonment, soil aggregation, soil degradation

## Abstract

Soil aggregate stability can indicate soil quality, and affects soil organic carbon (SOC) and soil organic nitrogen (SON) sequestration. However, for erodible soils, the effects of soil aggregate stability on SOC and SON under land use change are not well known. In this study, soil aggregate distribution, SOC and SON content, soil aggregate stability, and soil erodibility were determined in the soils at different depths along the stages following agricultural abandonment, including cropland, abandoned cropland, and native vegetation land in an erodible region of Southwest China. Soil aggregation, soil aggregate stability, and SOC and SON content in the 0–20 cm depth soils increased after agricultural abandonment, but soil texture and soil erodibility were not affected by land use change. Soil erodibility remained in a low level when SOC contents were over 20 g·kg^−1^, and it significantly increased with the loss of soil organic matter (SOM). The SOC and SON contents increased with soil aggregate stability. This study suggests that rapidly recovered soil aggregate stability after agricultural abandonment promotes SOM sequestration, whereas sufficient SOM can effectively maintain soil quality in karst ecological restoration.

## 1. Introduction

Agricultural abandonment is generally supposed to significantly increase the stocks of soil organic carbon (SOC) and soil organic nitrogen (SON) [1,2,3]. However, some studies report that the SOC and SON stocks show unaltered or decreased trends following agricultural abandonment, and are dependent on climate, prior land use type, and soil type [1,2]. In addition, many soil physical properties, for example, soil bulk density, porosity, and permeability, will be improved in the surface soils after agricultural abandonment [4], and these soil physical properties can affect the rate of soil organic matter (SOM) decomposition [5]. Soil microbial diversity and quantity generally increase after agricultural abandonment [6,7], which improves SOM stabilization through secretion of organic products. Increased vegetation abundance through agricultural abandonment aging enhances the input of SOM through leaf litters and root exudates [8]. The responses of the SOC and SON to land use change are largely unknown [3].

Soil aggregates are the basic unit of soil structure [9], and their stability is generally regarded as an indicator of soil quality. The mean weight diameter (MWD) of soil aggregates is often used to quantify soil aggregate stability [10].Soil aggregate stability is affected by a variety of binding agents, including organic materials, iron and aluminum oxides, carbonates, and metal cations [11]. In general, organic binding agents can significantly improve the water-stability of aggregates compared to inorganic binding agents [12]. Thus, these organic binding agents play important roles in resistance to soil erosion [13]. Stable aggregates provide a physical protection for SOM to reduce microbial attack [11]. Many studies in agroecosystems reported the distribution of soil aggregates, soil aggregate stability, and aggregate-associated SOC under different forms of land management, including no-tillage, fertilization, conversion of natural ecosystem into agriculture land, and vice versa [14,15,16,17,18]. Although soil aggregate stability and SOM sequestration are generally related phenomena [19], their interaction with land use change, including agricultural abandonment, remain unclear.

Soil degradation has been the greatest threat to ecological stability, residential environment, and local economic development in the karst region of Southwest China [20]. Soil degradation, including soil erosion and nutrient depletion, severely restricts agricultural productivity after long-term tillage [21,22]. Soil erodibility is defined as the amount of soil loss caused by runoff, rainfall, or seepage within a standard unit [23,24]. K factor is an estimated value from a soil erodibility estimate model, such as the Universal Soil Loss Equation (USLE), Revised Universal Soil Loss Equation (RUSLE), Erosion Productivity Impact Calculator (EPIC), or Geometric Mean Diameter based (Dg) model [23]. K factor is a key parameter for assessing the susceptibility of soil to erode, and partly reflects soil quality [23]. Previous studies have contrasted K factors in different erosion prediction models [23,24,25] and spatial variability, as affected by soil type, geology, geomorphology, climate, and agricultural management [26]. Soil erosion evolution in typical karst region was less than in Loess Plateau because of more complex geomorphology [26,27]. Few studies have examined the relationship between soil aggregate stability and SOM sequestration under land use change in karst-based highly erodible soils [28].

The objectives of this study were to (1) assess the changes in soil aggregate distribution, soil aggregate stability, SOC and SON content, and soil erodibility (K factor) at different stages after agricultural abandonment; and (2) determine the effects of soil aggregate stability on SOC and SON at different stages after agricultural abandonment of erodible soils in this region. These studies will improve the understanding of mechanistic changes in SOM sequestration and soil quality after agricultural abandonment. This will be of benefit through improving land management measures for ecological restoration and more sustainable soil productivity.

## 2. Materials and Methods 

### 2.1. Study Area

**Figure 1:**
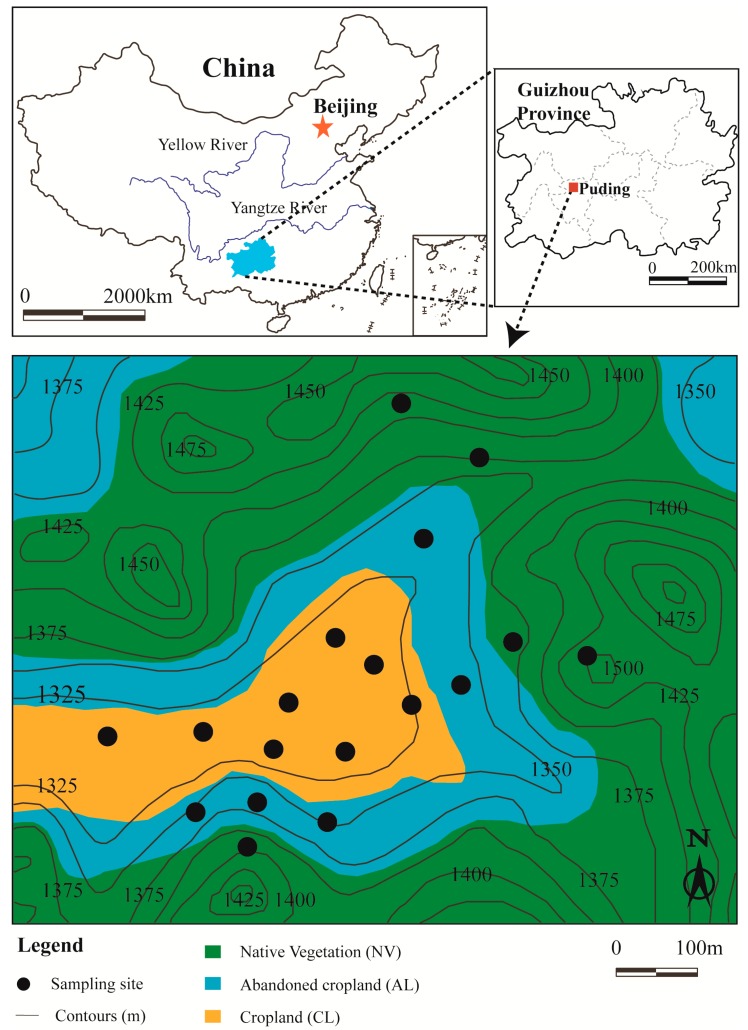
Location of study area and sampling sites.

The study sites (26°15′40″–26°16′10″ N, 105°46′02″–105°46′40″ E) were located in a karst region near Chenqi village in Puding county, Guizhou province, Southwest China (Figure 1). The region has a humid sub-tropical monsoon climate; the average annual air temperature is 15.1 °C; the mean annual precipitation is 1314.6 mm. More than 80% of annual precipitation occurs in the rainy season from April to September, while less than 20% occurs in the dry season from October to March [29,30]. The altitude of the study area ranges from 1310 to 1524 m, and an average altitude of 1350 m above sea level (a.s.l.) [31]. This region has a typical karst hoodoo depression landform; the center depression of the study area is surrounded by many hills, and over 60% of the hill areas have a gradient over 30° [32]. The lithology is mainly Permian and Triassic limestone and dolomite [33]. The calcareous soils are Mollic Inceptisols [34]. Soil layer thickness is spatially variable, ranging from 10 to 160 cm with an average of 30 cm [31]. The soils are distributed fragmentarily within the cavities and grooves between rocks, thus supporting the growth of crops and native plants.

In Chenqi, many arable soils have eroded along the cavities and grooves because of intensive agricultural production in the past 50 years [35]. Soil degradation, including both soil erosion and nutrient depletion, threatens the residential quality of life and the local economy [35]. In order to curtail soil erosion in the karst region, the government implemented the “Grain for Green Program” (GGP) at the beginning of the 21st century [23,36]. Many low yielding, steeply sloping croplands were abandoned, which were gradually covered by native vegetation [31]. In the study area, native plants are mainly thorn (*Itea yunnanensis*), hornbeam (*Carpinus pubescens)*, kembat (*Lithocarpus confinis*), and awn (*Miscanthus sinensis*) in shrub lands and secondary forest lands. Croplands have been cultivated over the long-term with tillage using moldboard plows and nutrient management using urea, ‘complete’ N-P-K fertilizers, and manure. Crops grown are peanut (*Arachis hypogaea)*, potato (*Solanum tuberosum*), oilseed rape (*Brassica napus*), and maize (*Zea mays*). Abandoned croplands are chiefly abandoned croplands and abandoned pear orchards with 3 to 8 years duration, and now have gradually evolved into grasslands and shrub lands.

### 2.2. Soil Sampling

In the study area, eight soil profiles from cropland (CL), five soil profiles from abandoned cropland (AL), and five soil profiles from native vegetation (NV) land were selected (Figure 1). The studies under three lands (CL, AL, and NV) were classified after agricultural abandonment (land use change) by space-for-time substitution approach [37]. Soil samples were collected from 0–10, 10–20, 20–30, 30–50, 50–70, and 70–90 cm depth soil layers, then air dried at room temperature.

### 2.3. Soil Analysis

Dried soil samples were separated into macro-aggregates (250–2000 μm), micro-aggregates (53–250 μm), and silt + clay sized fractions (<53 μm) by the modified method of wet sieving [31]. Macro-aggregates and micro-aggregates were collected after passing through 2000, 250, and 53 μm sifter in distilled water; silt + clay-sized fractions were separated using centrifugation. The moist aggregates were dried at 55 °C until constant weight, then weighed and the recovery calculated. These processes were produced three times, and the recovery of them remained 97–100%.

Soil samples were soaked in distilled water with soil:water of 1:2.5, stirred sufficiently and left to sit for 30 min until translucent suspensions [38]. The pH of the suspensions was measured using a PHS-3C pH-meter (Leici, Shanghai, China) with a precision of ± 0.05.

The organic bonding agents and calcareous cements between soil mineral particles were removed by hydrogen peroxide (H_2_O_2_) and by hydrochloric acid (HCl), respectively. The distribution of dispersive soil particles was measured by laser particle size analyzer (Mastersizer 2000, Malvern, England). Results were expressed as equivalent volume proportion with a precision of ± 1% [32]. Soil particles (0.05 mm < sand < 2 mm, 0.002 mm < silt < 0.05 mm, clay < 0.002 mm) were classified according to soil texture classification systems of the United States Department of Agriculture (USDA).

Dried soil samples were ground using agate mortar after removing big roots and stones, then passed through a 150 μm steel sifter. Carbonates were removed using 0.5 mol·L^−1^ HCl for 24 h [39]. Inorganic N (mainly including NO_3_^−^ and NH_4_^+^) was removed using 2 mol·L^−1^ potassium chloride (KCl) for 24 h [40]. Treated soil samples were centrifuged and washed with deionized water repeatedly until the supernatant liquids were neutral. The moist samples were dried at 55 °C until constant weight and ground into powder (<150 μm), which was used for analysis of SOC and SON content. The SOC and SON content was analyzed by combustion using a total organic carbon analyzer (Vario TOC Cube, Elementar, Germany) in the Laboratory of Surficial Environment Geochemistry, China University of Geosciences (Beijing). The reproducibility was monitored by replicate measurements using standard material low organic content soil (OAS B2152, C: 1.55 ± 0.04%; N: 0.13% ± 0.01%), and it was better than ± 0.1% for C and ± 0.02% for N.

### 2.4. Parameter Estimation

Measured SOC and SON contents should be calibrated because of loss of carbonate and inorganic N as follows:

SOC = SOC_m_ × M_1_/M_0_ × 10, (1)

SON = SON_m_ × M_1_/M_0_ × 10, (2)

where SOC and SON (g·kg^−1^) are actual content of organic C and N in soil, respectively; SOC_m_ and SON_m_ (%) are measured value in total organic carbon analyzer. M_0_ (g) is soil sample mass before removing carbonates and inorganic N; M_1_ (g) is mass after removing carbonates and inorganic N.

The mean weight diameter (*MWD*, mm) was calculated as [10]:

(3) $$\mathrm{MWD}\;=\;\overset{n}{\underset{k=1}{\sum }}X_{k}{\;\times \;M}_{k}$$

where *k* is aggregate size (*k* = 1, 2, 3 indicate macro-aggregate, micro-aggregate and silt + clay-sized fraction); $X_{k}$ (mm) is the mean diameter of the sized aggregate; and $M_{k}$ (%) is the mass proportion of the sized aggregate.

In the present study, soil erodibility factor (*Kepic*, t acre h (100·acre ft·tanf·in)^−1^) was estimated dependent on SOC content and soil particle size distribution based on EPIC mode [41] as

(4) $$\begin{array}{l} \mathrm{Kepic}\;=\left\{0.2\;+\;0.3\exp\left[-0.0256S\left(1\;-\;\frac{F}{100}\right)\right]\right\}\;\times {\left(\frac{F}{M\;+\;F}\right)}^{0.3}\\ \;\times \left[1.0\;-\frac{0.25C}{C\;+\;\exp(3.72-22.95C)}\right]\times \left[1.0\;-\;\frac{0.7E}{E\;+\;\exp\left(-5.51\;+\;22.9E\right)}\right] \end{array}$$

where *S*, *F*, *M* (%) represent the sand, silt, and clay contents, respectively; *E* (%) = 1–*S*/100; *C* (%) is SOC content. The *Kepic* as an American unit was translated into an international unit (t·ha·h (ha·MJ·mm)^−1^) by multiplying it by 0.1317.

In order to make the estimation of *K* factor in EPIC model with respect to Chinese soils, *Kepic* was adjusted according to the experiential formula as [42]

*K* = −0.01383 + 0.5158 *Kepic* (5)

For simplicity, the unit of K factor was omitted in this paper.

### 2.5. Statistical Analysis

One-way ANOVA analysis with least significant difference (LSD) test was performed to determine the significance of the stage after agricultural abandonment (land use change) on aggregate distribution, SOC content, SON content, MWD, and K factor at different land uses at the level of *p* < 0.05. The results were reported as the means (standard deviations), and the lowercases showed statistical differences of the compared means. Two-way ANOVA analysis with LSD test was used to examine the significance of land use change, soil depth, and their interactions on SOC content, SON content, MWD, and K factor. The relationships among SOC content, SON content, K factor, and MWD were determined by regression analyses; the best-fit lines were drawn, and coefficient *R^2^* and *p*-values were determined. Statistical analyses were performed using SPSS 18.0 (SPSS Inc., Chicago, IL, USA) software package. Figures were created using SigmaPlot 12.5 (Systat Software GmbH, Erkrath, Germany).

## 3. Results and Discussion

### 3.1. Soil Particle Distribution, Soil pH, and Aggregate Distribution under Different Land Uses

Distribution of clay, silt, and sand particles were almost constant along soil depth and different land use types (Table 1), which indicated that land use would not be expected to alter soil texture in this study area. Soil pH values at the same depth were not different under three land uses, but slightly increased with the increasing of soil depth (Table 1). More carbonates in topsoil were lost than that in subsoil via leaching, resulting in lower surface layer soil pH than in deeper layers. Soil particle distribution (clay: 17–19%; silt: 80–83%; sand: ~0) and soil pH (6.9–7.5) in the study area indicated a weak alkaline silt loam soil (USDA soil taxonomy), consistent with reports from other karst areas of Southwest China [43,44].

Macro-aggregate proportion under cropland was significantly lower than under abandoned cropland and native vegetation land at the 0–20 cm depth, but there were no significant differences among the three land uses in the soils below 20 cm depth (Table 2). Micro-aggregate proportions under abandoned cropland and native vegetation land were significantly lower than under cropland in the soils at the 0–30 cm depth (Table 2). Silt + clay-sized fraction proportion under cropland was larger than under abandoned cropland and native vegetation land in the soils at the 0–10 cm depth (Table 2). These results indicated that land use change can affect different-sized aggregates at different depth depended on aggregate size. Macro-aggregates accounted for 63–83% of all sized aggregates (Table 2). Furthermore, macro-aggregates were generally more sensitive to land management than smaller sized aggregates [45]. Therefore, only the changes in macro-aggregate proportion under different land uses and soil depth will be discussed with respect to the effects of land use and soil depth on soil aggregation. (1) Tillage in cropland destroyed macro-aggregate formation, which significantly reduced macro-aggregate proportion after long-term cultivation [15,46]. (2) Soil microbial biodiversity and quantity generally increased with recuperation of native vegetation after agricultural abandonment [6,7]. Soil microbial activity was key to macro-aggregate formation through various microbial secretions that acted as organic bonding agents between soil mineral particles [11]. (3) Agricultural abandonment often had more abundant vegetation from enhanced above-ground and underground plant biomass [8]. Larger above-ground and underground plant biomass in native vegetation land provided leaf litter and root exudates that can increase SOM. Concomitantly, abundant SOM improved microbial activity because of required energy substances for microbial metabolism. However, in the current study area, crop residues were not returned to soils in cropland, which restricted SOM increases and soil aggregation [47]. (4) Another consideration was that root growth promoted macro-aggregate formation by the binding of root exudations on clay particles and entanglement of particles by fine roots [11]. In this work, soils under native vegetation with larger plant biomass had more macro-aggregations formed. The macro-aggregate proportions in the 0–20 cm depth soils under cropland abandoned for 3 to 8 years was similar to those under native vegetation land, indicating the rapid rehabilitation ability of soil aggregation after agricultural abandonment. Previous work found about 90% of root biomass was in the top 20 cm soils in the karst region [48], and that land management, including tillage, generally affects <30 cm of soil depth. The effects of land use on soil aggregation were weak in the soils below 20 cm depth (Table 2) because of reduced root activity and lower SOM.

### 3.2. Effects of Land Use Change and Soil Depth on SOC, SON, Aggregate Stability, and K Factor

**Figure 2:**
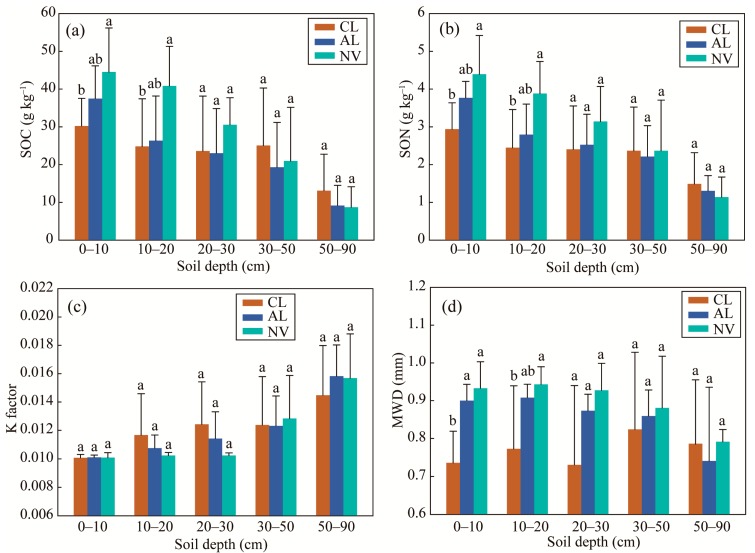
SOC content (**a**), SON content (**b**), K factor (**c**), and mean weight diameter (MWD) (**d**) in different depths of soil layer at different stages after agricultural abandonment. Lowercase letters indicate significant differences among the different stages after agricultural abandonment at *p* < 0.05 level on the basis of the least significant difference (LSD) test. NV, native vegetation land; AL, abandoned cropland; CL, cropland.

The SOC and SON contents under cropland were significantly lower than under native vegetation land at the 0–20 cm depth, and slightly increased under abandoned cropland (Figure 2a,b). As an important source of SOC and SON, SOM contents in surface soils mainly depend on dynamics between inputs of organic materials and microbial decomposition [49]. Leaf litter and root exudates associated with plant biomass contribute to SOM [8], but crop residues were not returned to soils in this study, which reduced SOM accumulation in cropland [47]. Tillage introduces oxygen into the soil and exposes the organic matters from deeper soils, accelerating SOM decomposition. SOM decomposition rate in abandoned cropland generally accelerates with increasing time because of increased soil biological activity [50]. In this study, plant biomass increased with the evolution of these croplands into grasslands and shrub lands, increasing SOM input and soil microbial activity. Soil microorganisms easily consume fresh and labile SOM, producing stable organic products [51,52] that increase stable SOC pool. Macro-aggregates provide physical isolation that protects the organic substrate from microbial attack [11]. Relatively lower macro-aggregate proportion in cropland soils restricts this physical protection, resulting a more rapid microbial decomposition. Significant increases in SOC and SON contents were not observed cropland abandoned for 3 to 8 years at the 0–20 cm depth, indicating that recovery rates of SOC and SON were slower than soil aggregation in these soils after agricultural abandonment. Significant differences of SOC and SON contents under different land uses were observed in 0–20 cm depth soils, and were likely related to root allocation and tillage disturbance in this soil layer [48].

The K factors were not different in the soils under native vegetation land, abandoned cropland, and cropland at the same soil depth (Figure 2c). In the soils under these land uses, the K factors increased with increasing of soil depth; however, an obvious increase occurred at 30 cm depth under native vegetation land, at 20 cm depth under abandoned cropland, and at 10 cm depth under cropland. These results suggested that land use change did not alter soil erodibility, but did affect the depth of measurable response in these soils. However, in the erodible area of the Loess Plateau of China, the abandonment of apple production (land use change) significantly affected soil erosion, and restoration age determined the affected depth [28].

The MWD under cropland was significantly lower than under abandoned cropland and native vegetation land at the 0–20 cm depth, whereas there were no significant differences among the three land uses in the soils below 20 cm depth (Figure 2d). Along land uses and soil depth, the variations of MWD were similar to the changes of macro-aggregate proportion, as macro-aggregate proportion (>63%) dominated. Essentially, soil aggregate stability is determined by binding agents between soil particles [53], and the organic materials are the dominant binding agents in these surface soils [31]. Therefore, the differences in plant biomass under different land uses [8] and unreturned crop residues in cropland [47] that affect organic material inputs and soil aggregate stability also affect MWD. As SOM sharply decreased with increasing soil depth, inorganic binding agents affected soil aggregate stability instead of organic binding agents. Soil aggregates bound by inorganic agents generally have less water stability than organic agents [12,54]. Thus, soil aggregate stability (i.e., MWD) in native vegetation land and abandoned cropland decreased with increasing soil depth. In cropland, increases of MWD at the layers below 30 cm depth compared to the 0–20 cm depth layers likely resulted from strong destruction to macro-aggregates under long-term tillage.

In this study, clay, silt, and sand contents were constant in the soils at different depths and under different land uses (Table 1); thus, soil texture would not be expected to affect K factor. Two-way ANOVA analysis with LSD test was used to identify the effects of soil depth; land use change; and their interaction on SOC content, SON content, K factor, and MWD (Table 3). Soil depth significantly affected SOC content, SON content, and K factor; however, there was no influence on MWD. Land use change significantly affected SOC content, SON content, and MWD, and did not affect K factor. SOC content, SON content, K factor, and MWD were not affected by the interaction of soil depth and land use change.

### 3.3. Effects of Aggregate Stability on SOC and SON in an Erodible Region

**Figure 3:**
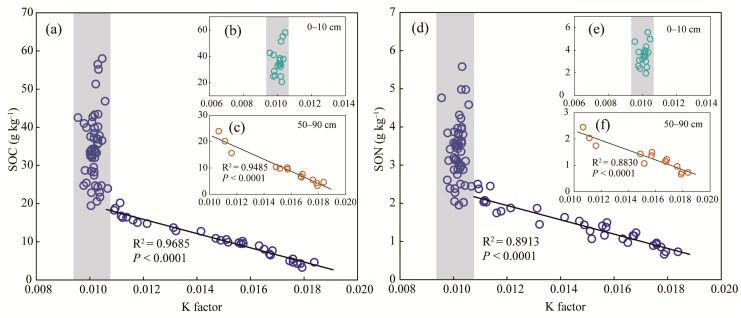
Relationships between K factor and SOC content in soils at all depths (**a**), in soils at 0–10 cm depth (**b**), and in soils at 50–90 cm depth (**c**); and relationships between K factor and SON content in soils at all depths (**d**), in soils at 0–10 cm depth (**e**), and in soils at 50–90 cm depth (**f**).

K factor was only affected by soil depth (Table 3); thus, the changes in K factor under different land use were ignored. K factors were consistent with values near 0.010 and did not vary with SOC content at the 0–10 cm depth (Figure 3b), whereas at 50–90 cm depths, a significant negative correlation between K factor and SOC content was observed (Figure 3c). For the soils at all depths, K factors remained constant values near 0.010 when SOC contents were relatively large (about >20 g·kg^−1^), and significantly increased with loss of SOC when SOC contents were below 20 g·kg^−1^ (Figure 3a). Similar relationships between K factor and SON content were observed (Figure 3d–f). These results suggest that sufficient SOM can sustain soil quality, and that SOM loss increases the risk of soil erosion. There were three main ways that SOM suppresses soil erodibility: (1) SOM absorbs soil water, which reduces detachment of soil particles from raindrop splash and runoff, and increases soil shear strength [55]; (2) Fine particles are immobilized by SOM through formations of allophane–Fe (Al)–OC complexes [51,52,56] and soil aggregates [11]; (3) SOM can maintain soil structure by increasing soil pore space, which can improve water infiltration.

**Figure 4:**
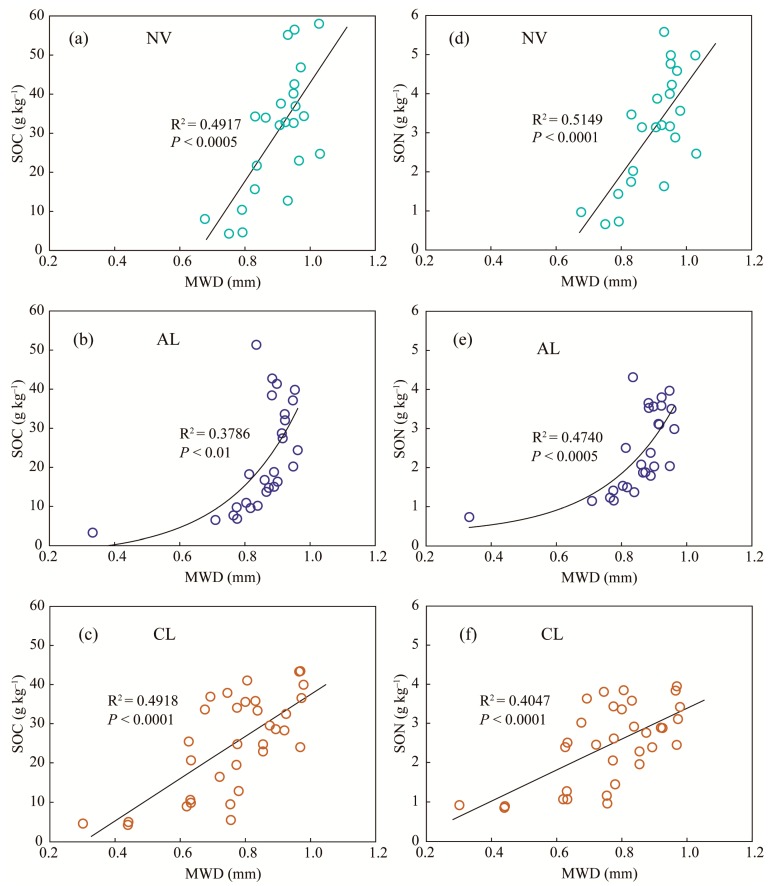
Relationships between MWD and SOC content in soils at all depths under native vegetation land (**a**), under abandoned cropland (**b**), and under cropland (**c**); and relationships between MWD and SON content in soils at all depths under native vegetation land (**d**), under abandoned cropland (**e**), and under cropland (**f**). NV, native vegetation land; AL, abandoned cropland; CL, cropland.

MWD was only affected by land use change in the present study (Table 3); thus, the changes in MWD at different depths were ignored. Regression analyses were used to examine the relationships between MWD (soil aggregate stability) and SOC content (or SON content) in the soils at all depths under native vegetation land, abandoned cropland, and cropland (Figure 4). Linear relationships between MWD and SOC content were observed in the soils under cropland and native vegetation land, and the slope of their fitting straight-line was significantly less in cropland soils than in native vegetation land soils (Figure 4a,c). However, an exponential relationship was observed in the soils under abandoned cropland, and the slope of tangent of the curve increased with increasing MWD (Figure 4b). The slopes described SOC content change along the gradient of soil aggregate stability, thus indicating the ability of SOC protection by soil aggregates. These results suggest that the soils under native vegetation land have greater potential to sequester SOC than those in the cropland soils due to increased soil aggregate stability, and that the potential in the soils under abandoned cropland can be recovered after agricultural abandonment. Similar relationships between MWD and SON content were observed (Figure 4d–f), indicating similar effects of soil aggregate stability on SON following agricultural abandonment.

Previously, positive correlations between MWD and SOM (or SOC) were observed in loess soils and bog soils [57,58], resulting from the dominant contribution of organic binding agents to soil aggregation [59,60]. In the present study, soil aggregation and SOM content in the surface soils under native vegetation land were significantly larger than those under cropland. Soil aggregates provide protection for SOM; meanwhile, SOM promotes macro-aggregate formation [11]. MWD was mainly determined by macro-aggregate proportion; thus, soil aggregation was associated with soil aggregate stability in this study. Hence, soil aggregate stability and SOM sequestration reinforced each other under the native vegetation land. In addition to increased oxidation, tillage also hindered macro-aggregate formation because SOM is more rapidly decomposed because of less aggregate-protection. In turn, decreased SOM restricts macro-aggregate formation [44]. Thus, soil aggregate stability and SOM sequestration were mutually inhibiting in the soils under the cropland. Therefore, the efficiency of SOM sequestration in the soils under native vegetation land was higher than in the cropland soils, and also increased soil aggregate stability. When tillage stopped in an abandonment situation, the surface covered by plant gradually increased. Thus, soil aggregate stability and SOM sequestration in abandoned cropland approached the levels in the soils under native vegetation. For the cropland abandoned for 3 to 8 years, the recovery of soil aggregate stability (or soil aggregation) in the surface soils was more rapid than the recuperation of SOM. SOM sequestration after agricultural abandonment is an important process because increasing soil aggregate stability enhances SOM protection.

## 4. Conclusions

SOC content in the surface soils under cropland (30 g·kg^−1^) was significantly lower than 45 g·kg^−1^ under native vegetation land, as well as SON content (2.9 g·kg^−1^/4.4 g·kg^−1^), macro-aggregate proportion (63%/82%), and MWD (0.73 mm/0.94 mm). Soil aggregation, soil aggregate stability, and SOC and SON content in surface soils increased following agricultural abandonment. For the most part, land use change would not be expected to alter soil texture and soil erodibility. Sufficient SOM (SOC content was over 20 g·kg^−1^) could effectively maintain soil quality (K factor remained at 0.01 t·ha·h (ha·MJ·mm)^−1^), and the risk of soil erosion would significantly increase with the loss in karst soils. The potential in SOC sequestration was gradually recovered after agricultural abandonment, because increased soil aggregate stability enhanced the protection for SOM. This study suggests that soil aggregation can rapidly recover after agricultural abandonment, which is critical for recuperation of soil quality, restraining of soil erosion, and reduction of nutrient loss to other ecosystems in karst agroecosystems.

## Figures and Tables

**Table 1:** Soil particle distribution and soil pH at different stages after agricultural abandonment.

Soil Depth (cm)	Clay Content (%)			Silt Content (%)			Sand Content (%)			Soil pH		
	NV	AL	CL	NV	AL	CL	NV	AL	CL	NV	AL	CL
0–10	18 (3)	18 (2)	20 (3)	81 (4)	81 (2)	80 (3)	1 (2)	–	–	7.2 (0.6)	7.0 (0.3)	6.9 (0.5)
10–20	17 (2)	19 (1)	20 (3)	83 (2)	81 (1)	80 (3)	–	–	–	7.3 (0.4)	7.1 (0.4)	7.1 (0.5)
20–30	18 (2)	19 (2)	18 (2)	82 (2)	81 (2)	82 (2)	–	–	–	7.4 (0.4)	7.2 (0.4)	7.2 (0.4)
30–50	17 (3)	19 (2)	19 (2)	83 (3)	81 (2)	81 (2)	–	–	–	7.4 (0.2)	7.2 (0.6)	7.2 (0.2)
50–90	19 (4)	19 (4)	19 (5)	81 (4)	81 (4)	81 (5)	–	–	–	7.5 (0.1)	7.2 (0.6)	7.4 (0.1)

Note: Expressed as mean (standard deviation). NV, native vegetation land; AL, abandoned cropland; CL, cropland. “–”indicates that the value was close to 0.

**Table 2:** Distribution of different sized aggregates at different stages after agricultural abandonment.

Soil Depth (cm)	Macro-Aggregate (%)			Micro-Aggregate (%)			Silt + Clay-Sized Fraction (%)		
	NV	AL	CL	NV	AL	CL	NV	AL	CL
0–10	82 (7) ^a^	79 (4) ^a^	63 (8) ^b^	8 (2) ^b^	9 (2) ^b^	15 (3) ^a^	10 (5) ^b^	12 (2) ^b^	22 (7) ^a^
10–20	83 (4) ^a^	79 (3) ^ab^	66 (16) ^b^	8 (2) ^b^	9 (3) ^ab^	15 (6) ^a^	10 (3) ^a^	12 (2) ^a^	19 (11) ^a^
20–30	81 (7) ^a^	76 (4) ^a^	62 (19) ^a^	7 (2) ^b^	10 (1) ^ab^	15 (5) ^a^	11 (6) ^a^	14 (3) ^a^	23 (17) ^a^
30–50	77 (17) ^a^	75 (6) ^a^	71 (19) ^a^	9 (2) ^a^	10 (1) ^a^	12 (5) ^a^	15 (13) ^a^	16 (5) ^a^	16 (14) ^a^
50–90	68 (3) ^a^	64 (18) ^a^	67 (16) ^a^	13 (2) ^a^	12 (3) ^a^	16 (8) ^a^	19 (4) ^a^	24 (18) ^a^	16 (9) ^a^

Note: Expressing as mean (standard deviation). Lowercase letters indicate significant differences among the different stages after agricultural abandonment at *p* < 0.05 level on the basis of the least significant difference (LSD) test. NV, native vegetation land; AL, abandoned cropland; CL, cropland.

**Table 3:** Effects of soil depth, land use change, and their interactions on soil organic carbon (SOC) content, soil organic nitrogen (SON) content, K factor, and MWD.

Factor	Variable	df	*F*	Sig.
Soil depth	SOC	4	12.038	0
	SON	4	14.186	0
	K factor	4	11.845	0
	MWD	4	1.198	0.32 ns
Land use change	SOC	2	2.296	0.108
	SON	2	3.523	0.035
	K factor	2	0.199	0.82 ns
	MWD	2	6.17	0.003
Soil depth × land use change	SOC	8	1.058	0.403 ns
	SON	8	1.103	0.372 ns
	K factor	8	0.543	0.82 ns
	MWD	8	0.709	0.682 ns

Note: ns indicates non-significant differences.

## References

[1] Deng L., Liu G.B., Shangguan Z.P. (2014). Land-use conversion and changing soil carbon stocks in China’s ’Grain-for-Green’ Program: A synthesis. Glob. Chang. Biol..

[2] Li D., Niu S., Luo Y. (2012). Global patterns of the dynamics of soil carbon and nitrogen stocks following afforestation: A meta-analysis. New Phytol..

[3] Li D., Wen L., Yang L., Luo P., Xiao K., Chen H., Zhang W., He X., Chen H., Wang K. (2017). Dynamics of soil organic carbon and nitrogen following agricultural abandonment in a karst region. J. Geophys. Res..

[4] Cavalieri K.M.V., Silva A.P.D., Tormena C.A., Leão T.P., Dexter A.R., Håkansson I. (2009). Long-term effects of no-tillage on dynamic soil physical properties in a Rhodic Ferrasol in Paraná, Brazil. Soil Tillage Res..

[5] Dungait J.A.J., Hopkins D.W., Gregory A.S., Whitmore A.P. (2012). Soil organic matter turnover is governed by accessibility not recalcitrance. Glob. Chang. Biol..

[6] Li D., Zhang X., Green S.M., Dungait J.A.J., Wen X., Tang Y., Guo Z., Yang Y., Sun X., Quine T.A. (2018). Nitrogen functional gene activity in soil profiles under progressive vegetative recovery after abandonment of agriculture at the Puding Karst Critical Zone Observatory, SW China. Soil Biol. Biochem..

[7] Chavarria D.N., Pérez-Brandan C., Serri D.L., Meriles J.M., Restovich S.B., Andriulo A.E., Jacquelin L., Vargas-Gil S. (2018). Response of soil microbial communities to agroecological versus conventional systems of extensive agriculture. Agric. Ecosyst. Environ..

[8] Zhang J., Zhao H., Zhang T., Zhao X., Drake S. (2005). Community succession along a chronosequence of vegetation restoration on sand dunes in Horqin Sandy Land. J. Arid Environ..

[9] Lynch J., Bragg E. (1985). Microorganisms and soil aggregate stability. Advances in Soil Science.

[10] Choudhury S.G., Srivastava S., Singh R., Chaudhari S.K., Sharma D.K., Singh S.K., Sarkar D. (2014). Tillage and residue management effects on soil aggregation, organic carbon dynamics and yield attribute in rice-wheat cropping system under reclaimed sodic soil. Soil Tillage Res..

[11] Six J., Bossuyt H., Degryze S., Denef K. (2004). A history of research on the link between (micro)aggregates, soil biota, and soil organic matter dynamics. Soil Tillage Res..

[12] Tisdall J.M., Oades J.M. (1982). Organic matter and water-stable aggregates in soils. Eur. J. Soil Sci..

[13] Annabi M., Raclot D., Bahri H., Bailly J.S., Gomez C., Bissonnais Y.L. (2017). Spatial variability of soil aggregate stability at the scale of an agricultural region in Tunisia. Catena.

[14] Six J., Elliott E., Paustian K. (1999). Aggregate and soil organic matter dynamics under conventional and no-tillage systems. Soil Sci. Soc. Am. J..

[15] Six J., Elliott E.T., Paustian K., Doran J.W. (1998). Aggregation and Soil Organic Matter Accumulation in Cultivated and Native Grassland Soils. Soil Sci. Soc. Am. J..

[16] Garland G., Bünemann E.K., Oberson A., Frossard E., Six J. (2017). Plant-mediated rhizospheric interactions in maize-pigeon pea intercropping enhance soil aggregation and organic phosphorus storage. Plant Soil.

[17] Jiang M., Wang X., Liusui Y., Chao H., Zhao C., Hua L. (2017). Variation of soil aggregation and intra-aggregate carbon by long-term fertilization with aggregate formation in a grey desert soil. Catena.

[18] Bhattacharyya R., Prakash V., Kundu S., Srivastva A.K., Gupta H.S., Mitra S. (2010). Long term effects of fertilization on carbon and nitrogen sequestration and aggregate associated carbon and nitrogen in the Indian sub-Himalayas. Nutr. Cycl. Agroecosyst..

[19] Villasica L.J., Lina S., Asio V. (2018). Aggregate stability affects carbon sequestration potential of different tropical soils. Ann. Trop. Res..

[20] Zhou W., Xie S., Zhu L., Tian Y., Jia Y. (2008). Evaluation of soil erosion risk in Karst regions of Chongqing, China. SPIE.

[21] Zeng J., Yue F.J., Wang Z.J., Wu Q., Qin C.Q., Li S.L. (2019). Quantifying depression trapping effect on rainwater chemical composition during the rainy season in karst agricultural area, southwestern China. Atmos. Environ..

[22] Deng L., Zhang Z., Shangguan Z. (2014). Long-term fencing effects on plant diversity and soil properties in China. Soil Tillage Res..

[23] Wang B., Zheng F., Römkens M.J.M., Darboux F. (2013). Soil erodibility for water erosion: A perspective and Chinese experiences. Geomorphology.

[24] Wang B., Zheng F., Guan Y. (2016). Improved USLE-K factor prediction: A case study on water erosion areas in China. Int. Soil Water Conserv. Res..

[25] Zhang K., Yu Y., Dong J., Yang Q., Xu X. (2019). Adapting & testing use of USLE K factor for agricultural soils in China. Agric. Ecosyst. Environ..

[26] Ye L., Tan W., Fang L., Ji L., Deng H. (2018). Spatial analysis of soil aggregate stability in a small catchment of the Loess Plateau, China: I. Spatial variability. Soil Tillage Res..

[27] Zeng C., Wang S., Bai X., Li Y., Tian Y., Li Y., Wu L., Luo G. (2017). Soil erosion evolution and spatial correlation analysis in a typical karst geomorphology, using RUSLE with GIS. Solid Earth.

[28] Zhu G., Deng L., Shangguan Z. (2018). Effects of soil aggregate stability on soil N following land use changes under erodible environment. Agric. Ecosyst. Environ..

[29] Zhao M., Zeng C., Liu Z.H., Wang S.J. (2010). Effect of different land use/land cover on karst hydrogeochemistry: A paired catchment study of Chenqi and Dengzhanhe, Puding, Guizhou, SW China. J. Hydrol..

[30] Zhang Q., Han G., Liu M., Wang L. (2019). Geochemical Characteristics of Rare Earth Elements in Soils from Puding Karst Critical Zone Observatory, Southwest China. Sustainability.

[31] Liu M., Han G., Li Z., Zhang Q., Song Z. (2019). Soil organic carbon sequestration in soil aggregates in the karst Critical Zone Observatory, Southwest China. Plant Soil Environ..

[32] Liu M., Han G., Zhang Q., Song Z. (2019). Variations and Indications of δ^13^C_SOC_ and δ^15^N_SON_ in Soil Profiles in Karst Critical Zone Observatory (CZO), Southwest China. Sustainability.

[33] Zhang Q., Han G., Liu M., Liang T. (2019). Spatial distribution and controlling factors of heavy metals in soils from Puding Karst Critical Zone Observatory, southwest China. Environ. Earth Sci..

[34] Staff S.S. (2010). Keys to Soil Taxonomy.

[35] Wang S.J., Liu Q.M., Zhang D.F. (2004). Karst rocky desertification in southwestern China: Geomorphology, landuse, impact and rehabilitation. Land Degrad. Dev..

[36] Wang B., Gao P., Niu X., Sun J. (2017). Policy-driven China’s Grain to Green Program: Implications for ecosystem services. Ecosyst. Serv..

[37] Blois J.L., Williams J.W., Fitzpatrick M.C., Jackson S.T., Ferrier S. (2013). Space can substitute for time in predicting climate-change effects on biodiversity. Proc. Natl. Acad. Sci. USA.

[38] Liu G.S., Jiang N.H., Zhang L.D., Liu Z.L. (1996). Soil Physical and Chemical Analysis and Description of Soil Profiles.

[39] Midwood A.J., Boutton T.W. (1998). Soil carbonate decomposition by acid has little effect on δ^13^C of organic matter. Soil Biol. Biochem..

[40] Meng L., Ding W., Cai Z. (2005). Long-term application of organic manure and nitrogen fertilizer on N_2_O emissions, soil quality and crop production in a sandy loam soil. Soil Biol. Biochem..

[41] Sharpley A.N., Williams J.R. (1990). EPIC-erosion/productivity impact calculator: 1. Model determination. USDA Technol. Bull..

[42] Zhang K.L., Shu A.P., Xu X.L., Yang Q.K., Yu B. (2008). Soil erodibility and its estimation for agricultural soils in China. J. Arid Environ..

[43] Han G., Song Z., Tang Y. (2017). Geochemistry of rare earth elements in soils under different land uses in a typical karst area, Guizhou Province, Southwest China. Can. J. Soil Sci..

[44] Han G., Li F., Tang Y. (2015). Variations in soil organic carbon contents and isotopic compositions under different land uses in a typical karst area in Southwest China. Geochem. J..

[45] Franzluebbers A.J., Arshad M.A. (1997). Soil Microbial Biomass and Mineralizable Carbon of Water-Stable Aggregates. Soil Sci. Soc. Am. J..

[46] Six J., Elliott E.T., Paustian K. (2000). Soil macroaggregate turnover and microaggregate formation: A mechanism for C sequestration under no-tillage agriculture. Soil Biol. Biochem..

[47] Liu M., Han G.L., Li Z.C., Liu T.Z., Yang X.M., Wu Y.T., Song Z.L. (2017). Effects of slope position and land use on the stability of aggregate-associated organic carbon in calcareous soils. Acta Geochim..

[48] Ni J., Luo D.H., Xia J., Zhang Z.H., Hu G. (2015). Vegetation in karst terrain of southwestern China allocates more biomass to roots. Solid Earth.

[49] Schlesinger W.H. (1977). Carbon balance in terresterial detritus. Ann. Rev. Ecol. Syst..

[50] Houben D., Faucon M.P., Mercadal A.M. (2018). Response of Organic Matter Decomposition to No-Tillage Adoption Evaluated by the Tea Bag Technique. Soil Syst..

[51] Aiken G.R., Mcknight D.M., Wershaw R.L., Maccarthy P. (1985). Humic Substances in Soil, Sediment, and Water: Geochemistry, Isolation and Characterization.

[52] Stevenson F.J. (1991). Organic matter-micronutrient reactions in soil. Micronutrients in Agriculture.

[53] Oades J.M., Waters A.G. (1991). Aggregate hierarchy in soils. Aust. J. Soil Res..

[54] Oades J.M. (1984). Soil organic matter and structural stability: Mechanisms and implications for management. Plant Soil.

[55] Zhu B., Li Z., Li P., Liu G., Xue S. (2010). Soil erodibility, microbial biomass, and physical–chemical property changes during long-term natural vegetation restoration: A case study in the Loess Plateau, China. Ecol. Res..

[56] Rodríguez Rodríguez A., Arbelo C.D., Guerra J.A., Mora J.L., Notario J.S., Armas C.M. (2006). Organic carbon stocks and soil erodibility in Canary Islands Andosols. Catena.

[57] Spohn M., Giani L. (2011). Impacts of land use change on soil aggregation and aggregate stabilizing compounds as dependent on time. Soil Biol. Biochem..

[58] Zeng Q., Darboux F., Man C., Zhu Z., An S. (2018). Soil aggregate stability under different rain conditions for three vegetation types on the Loess Plateau (China). Catena.

[59] Haynes R.J., Swift R.S., Stephen R.C. (1991). Influence of mixed cropping rotations (pasture-arable) on organic matter content, water stable aggregation and clod porosity in a group of soils. Soil.Tillage Res..

[60] Shepherd T.G., Saggar S., Newman R.H., Ross C.W., Dando J.L. (2001). Tillage-induced changes to soil structure and organic carbon fractions in New Zealand soils. Aust. J. Soil Res..

